# New insights into the activities and toxicities of the old anticancer drug doxorubicin

**DOI:** 10.1111/febs.15583

**Published:** 2020-10-19

**Authors:** Sabina Y. van der Zanden, Xiaohang Qiao, Jacques Neefjes

**Affiliations:** ^1^ Department of Cell and Chemical Biology ONCODE Institute Leiden University Medical Centre LUMC The Netherlands; ^2^ Division of Tumour Biology and Immunology The Netherlands Cancer Institute Amsterdam The Netherlands; ^3^ Department of Head and Neck Oncology and Surgery The Netherlands Cancer Institute Amsterdam The Netherlands

**Keywords:** aclarubicin, anthracyclines, cancer, cardiotoxicity, chromatin damage, DNA damage, doxorubicin, histone eviction, therapy‐related tumours, topoisomerase II

## Abstract

The anthracycline drug doxorubicin is among the most used—and useful—chemotherapeutics. While doxorubicin is highly effective in the treatment of various hematopoietic malignancies and solid tumours, its application is limited by severe adverse effects, including irreversible cardiotoxicity, therapy‐related malignancies and gonadotoxicity. This continues to motivate investigation into the mechanisms of anthracycline activities and toxicities, with the aim to overcome the latter without sacrificing the former. It has long been appreciated that doxorubicin causes DNA double‐strand breaks due to poisoning topoisomerase II. More recently, it became clear that doxorubicin also leads to chromatin damage achieved through eviction of histones from select sites in the genome. Evaluation of these activities in various anthracycline analogues has revealed that chromatin damage makes a major contribution to the efficacy of anthracycline drugs. Furthermore, the DNA‐damaging effect conspires with chromatin damage to cause a number of adverse effects. Structure–activity relationships within the anthracycline family offer opportunities for chemical separation of these activities towards development of effective analogues with limited adverse effects. In this review, we elaborate on our current understanding of the different activities of doxorubicin and their contributions to drug efficacy and side effects. We then offer our perspective on how the activities of this old anticancer drug can be amended in new ways to benefit cancer patients, by providing effective treatment with improved quality of life.

## Graphical abstract

The anthracycline drug doxorubicin is an effective anticancer drugs with both DNA‐ and chromatin‐damaging activity. While the chromatin‐damaging activity constitutes major anticancer efficacy of doxorubicin, combination with the DNA‐damaging activity plagues the drug with long‐term toxicities such as cardiotoxicity, therapy‐related malignancies and gonadotoxicity. Therefore, developing DNA damage‐free anthracyclines is a promising direction for novel treatment options with limited side effects, which has been shown to be possible.

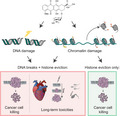

**Abbreviations**
AML: acute myeloid leukaemia
APL: acute promyelocytic leukaemia
DAMPs: damage‐associated molecular patterns
DSBs: double‐strand breaks
H_2_O_2_
: hydrogen peroxide
ICD: immunogenic cell death
diMe‐doxorubicin: *N*,*N*‐dimethyl‐doxorubicin
$O_{2}^{\cdot ‐}$
: superoxide anion
ROS: reactive oxygen species
t‐AML: therapy‐related acute myeloid leukaemia
t‐APL: therapy‐related acute promyelocytic leukaemia
t‐MNs: therapy‐related malignant neoplasms
Topo II: topoisomerase II
Topo IIα: topoisomerase II alpha
Topo IIβ: topoisomerase II beta

## Introduction

**Fig. 1:**
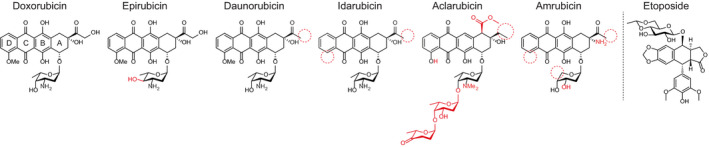
Structures of different anthracycline drugs and the structurally unrelated Topo II poison etoposide. Aglycon rings are numbered in doxorubicin. Structural differences compared with doxorubicin are indicated in red.

Doxorubicin, also known as adriamycin, is a member of the anthracycline anticancer drug family (Fig. 1). The first anthracycline drug, daunorubicin, was isolated from a soil sample found in Italy in 1960 [1, 2]. Daunorubicin is a pigmented antibiotic produced by the actinobacterium strain *Streptomyces peucetius* [2]. Soon, it was discovered that daunorubicin displayed anticancer activity in mice, which spurred its clinical use for the treatment of leukaemia, lymphoma and solid tumours in the late 1960s [3, 4]. In 1969, a daunorubicin homologue, doxorubicin, was isolated from a culture of chemically mutated *S. peucetius* [5]. Doxorubicin showed an even broader anticancer activity than daunorubicin, especially against solid tumours [6, 7]. However, quickly a major side effect of both otherwise highly potent anticancer drugs was noted—cardiotoxicity [8]. Cardiotoxicity incited by anthracyclines develops in a dose‐dependent manner and can be lethal [9, 10]. As a result, treatment has to be stopped once the maximal tolerated cumulative dose is reached, while patients with poor heart function are excluded from chemo regimens containing anthracyclines. In addition to treatment‐limiting cardiotoxicity, therapy‐related malignancies and gonadotoxicity are also associated with anthracycline treatment [9, 11]. With latest improvements in cancer therapy, the emphasis in cancer management has changed from ‘cure at any cost’ to giving quality of life after treatment more consideration. In this light, quests to understand and alleviate the side effects incurred by anthracyclines have been revived. Here, we provide an overview of the mechanisms of action and toxicity of anthracycline drugs and discuss different attempts that have been made to improve them. This is followed by our perspective on how to detoxify doxorubicin for effective anticancer treatment with limited adverse effects.

## Mechanisms of action of anthracycline drugs

### Topoisomerase II poison

**Fig. 2:**
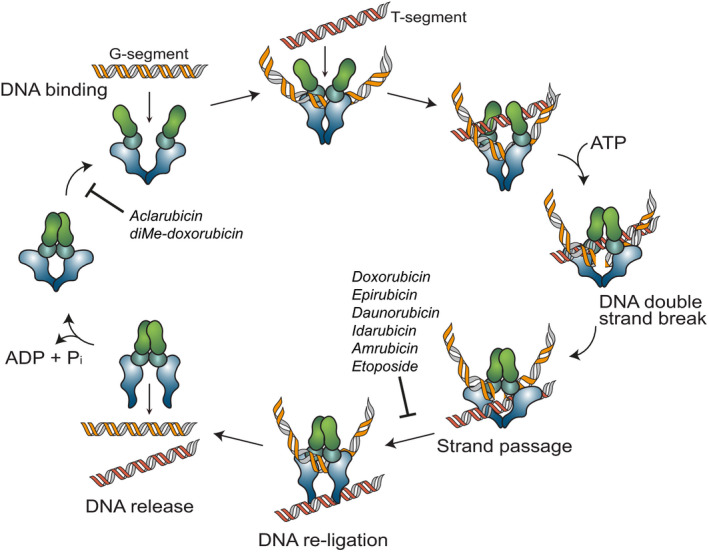
Schematic representation of the Topo II poisoning mechanism of anthracyclines. To entangle DNA or to remove DNA supercoils Topo II binds to DNA, introduce a transient DSB in one of the DNA strands (the G‐segment), allowing the second DNA strand (the T‐segment) to pass through. After re‐ligation of the G‐segment, the Topo II is released from DNA [15, 16, 17, 18]. The majority of Topo II poisons, including most anthracyclines (doxorubicin, daunorubicin, epirubicin, idarubicin and amrubicin) and etoposide, stabilize the Topo II complex after it has introduced the DNA DSB and prevent the DNA break from being resealed [13, 26]. Anthracycline variants aclarubicin and diMe‐doxorubicin inhibit the enzymatic activity by preventing Topo II from loading onto the DNA [13, 44]. Figure is inspired by [13].

The classical mechanism of action by which anthracyclines function is inhibition or poisoning of Topoisomerase II (Topo II) [12]. This enzyme plays a critical role in chromosome condensation, decatenation of intertwined DNA strands and relaxation of tension in the DNA strand in front of the replication fork [13, 14]. Topo II acts by introducing a transient double‐strand break (DSB) in one DNA strand (the G‐segment), allowing another DNA strand (the T‐segment) to pass through and subsequently closing the initial break by re‐ligation of the two DNA ends (Fig. 2) [13, 14, 15, 16, 17, 18]. Most anthracyclines (e.g. doxorubicin, epirubicin, daunorubicin, idarubicin, and amrubicin) intercalate into DNA and poison Topo II in its catalytic step following initial break induction by forming Topo II‐DNA complexes. These anthracyclines interfere at the interface of Topo II‐DNA with their sugar moieties and the cyclohexane ring A [19]. In essence, the interfacial positioning makes these anthracyclines act as molecular doorstops and prevent Topo II from re‐ligating the broken strand, which ultimately results in enzyme‐mediated DNA damage in the form of DSB [12, 20, 21]. Although the protein structure of a Topo II‐DNA‐doxorubicin complex is not available (reason will be discussed in the latter part), the door‐stopping act of doxorubicin can be deduced from the structure of a counterpart complex with the nonanthracycline Topo II poison, etoposide [22, 23, 24]. As a consequence of DSBs, DNA damage response (DDR) and TP53 pathways are activated, which lead to cell cycle arrest and cell death [25]. Some anthracyclines interrupt Topo II at other steps of the catalytic cycle, such as preventing the enzyme binding to the DNA (e.g. aclarubicin) or inhibiting ATP binding [13]. Topo II is essential for the survival of rapidly dividing cells, such as cancer cells that are more sensitive to DNA breaks than normal quiescent cells; hence, anthracyclines create a chemotherapeutic window by hijacking the essential enzyme function in cells [26]. For the same reason, anthracyclines also cause side effects, such as hair loss, bone marrow suppression and gastrointestinal complications.

### DNA intercalation

Anthracyclines intercalate into the DNA helix with their anthraquinone moiety. While rings B and C of the tetracycline moiety overlap with adjacent DNA base pairs, and ring D passes through the intercalation site, the sugar moiety is pointed into the minor groove, which may compete for space with histones [19, 27]. In addition to stabilizing the Topo II‐DNA complex, DNA intercalation of anthracyclines has additional effects, such as inhibiting DNA and RNA synthesis [28, 29].

### Oxidative stress

The quinone moiety in ring C of anthracyclines can be transformed into a semiquinone by a number of oxidoreductases, including cytochrome P450 reductases, xanthine oxidase and NADH dehydrogenase (complex I) of the mitochondrial electron transport chain [30, 31]. Subsequently, this semiquinone quickly regenerates and thereby converts oxygen into reactive oxygen species (ROS), such as superoxide anion ($O_{2}^{\cdot ‐}$) and hydrogen peroxide (H_2_O_2_), or oxidize the bond between the sugar and the aglycon resulting in reductive deglycosylation. Eventually $O_{2}^{\cdot ‐}$, and H_2_O_2_ are converted into more reactive hydroxyl radicals (^·^OH) via the iron‐catalysed Haber‐Weiss reaction [32, 33]. In addition, anthracyclines can also mediate ROS production by directly interfering with iron metabolism. They can increase cellular levels of iron by interacting with iron regulatory proteins (IRP1 and/or IRP2) or accelerate the release of iron from ferritin, which then further amplifies iron‐mediated oxidative stress [34, 35, 36]. The excessive ROS production can lead to lipid oxidation, genomic and mitochondrial DNA damage, which are toxic to cells. Nevertheless, the contribution of ROS formation to the anticancer activity of anthracyclines is still unclear and heavily discussed. It is worth noting that excessive ROS production is often observed when cells were exposed to anthracycline doses that are much higher than clinical relevant concentrations. Yet, at physiological concentrations, significant ROS formation was observed at late time points after drug removal, indicating this might be a secondary effect of anthracycline treatment rather than a direct mode of action [37]. Notwithstanding, it cannot be excluded that ROS formation may reinforce other mechanisms of anthracyclines.

### Chromatin damage

**Fig. 3:**
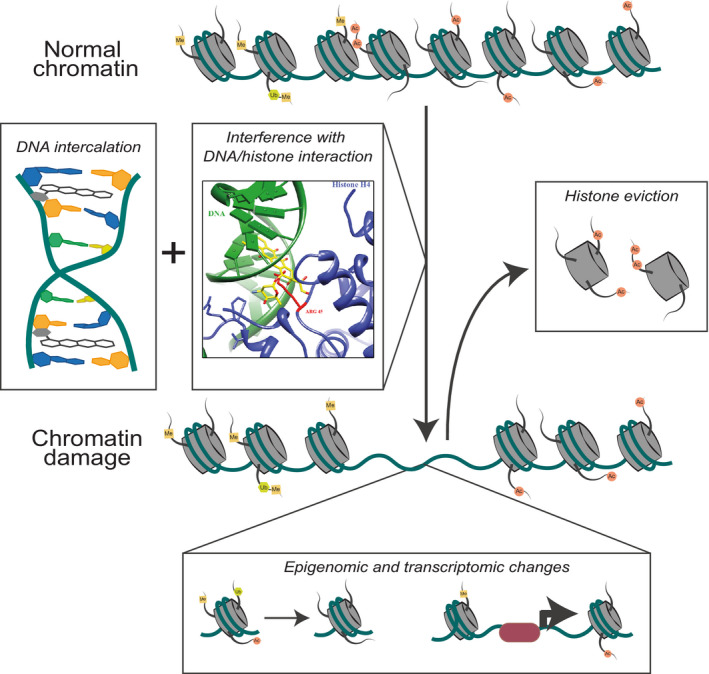
Schematic representation of chromatin damage induced by doxorubicin. Besides DNA intercalation by its anthraquinone group, doxorubicin's sugar moiety destabilizes nucleosome by competing for space with histones. Histone eviction caused by doxorubicin is shown to be ATP‐, transcription‐, and histone chaperone‐independent [27]. Histone eviction results in epigenetic and transcriptomic alterations and DSB repair attenuation, collectively referred to as chromatin damage. Part of the figure is reproduced from [27].

To organize two metres of DNA in the nucleus of a single cell, DNA is compacted at several levels. One level of organization is the formation of nucleosomes, where a segment of 146 base pairs of DNA is wrapped around eight histone proteins [38]. As mentioned above, when an anthracycline intercalates into DNA, the sugar moiety emanates into the DNA minor groove and competes with histones for space, resulting in the collapse of nucleosomes. As a result, histones are evicted from chromatin (Fig. 3) [27, 39]. *In vitro* experiments with reconstituted single nucleosomes showed that doxorubicin causes nucleosome dissociation in an ATP‐, transcription‐ and histone chaperone‐independent manner, which may explain why the structure of Topo II‐DNA‐doxorubicin complex is not available [27]. Moreover, the doxorubicin metabolite doxorubicinone, which lacks the sugar moiety of doxorubicin, was not able to dissociate nucleosomes under the same condition, suggesting a critical contribution of the sugar moiety to histone eviction [27, 40]. These data indicate that histone eviction induced by anthracyclines is a drug intrinsic process, which is cooperatively mediated by DNA intercalation of the anthraquinone group and nucleosome destabilization by the sugar moiety. This unique activity is not observed for other DNA intercalators (e.g. ethidium bromide [27]) or other chemotherapeutics (e.g. amsacrine or proflavin, data not published).

The dynamic structure of chromatin is essential for many nuclear processes, including transcription and replication. Therefore, the assembly, spatial organizing and compactization of chromatin are tightly regulated by various histone chaperones, ATP‐dependent chromatin remodelling complexes and histone‐modifying enzymes [41, 42]. Being the building blocks of chromatin, histones are directly involved in the regulation of these processes via different epigenetic modifications. Upon eviction, these modified histones are replaced by new/nascent ones with less or different epigenetic marks. This results in DDR delay, epigenetic and transcriptomic alterations, collectively termed as chromatin damage [43]. With the aid of next‐generation sequencing, unbiased (epi)genomic analysis revealed that each anthracycline evicts histones at select (epi)genomic regions [27, 43]. More specifically, doxorubicin evicts histones at open genomic regions marked by H3K36me3; while aclarubicin, whose sugar moiety is different from doxorubicin, induces histone eviction in a wider range, including compacted chromatin regions decorated by H3K27me3. As a matter of fact, anthracyclines could therefore be considered as epigenetic modifiers with defined (epi)genomic selectivity.

How histone eviction exactly causes cell death remains unclear, but it is likely to play a major contribution to the anticancer activity of the anthracycline drugs. This is illustrated by the anthracycline drugs aclarubicin and *N,N*‐dimethyl‐doxorubicin (diMe‐doxorubicin), which induce histone eviction without generating DNA damage [44]. Aclarubicin is prescribed mainly for the treatment of acute myeloid leukaemia (AML), showing similar efficacy as doxorubicin [27, 39, 44]. While aclarubicin was once used worldwide, it is currently only used in Japan and China. The specific reason behind this is not clear, and there are no clinical data that can explain the halt of usage. On the other hand, the doxorubicin analogue diMe‐doxorubicin was first reported in the 1980s [45]. It exhibited similar anticancer activity compared with doxorubicin in tissue culture experiments and in mice [27, 39, 44]. Further, its pharmacokinetics was tested in mice and rabbits [46], but no further follow‐up was reported. Surprisingly, it was recently shown by our laboratory that diMe‐doxorubicin only induces chromatin damage but no DSB, suggesting that chromatin damage rather that DNA breaks may be the dominant cytotoxic mechanism [44]. This is further substantiated by the anthracycline variant amrubicin, which only induces DSBs. Amrubicin is much less effective than doxorubicin, aclarubicin and diMe‐doxorubicin in killing cancer cells and thus did not enter clinic. Taken together, this implies that chromatin damage rather than DSB formation constitutes the major anticancer activity of anthracyclines.

### Immune modulation

Besides the direct effect on eliminating tumour cells, anthracyclines can also promote antitumour immunity. During cell death, cell contents can be released into the tumour microenvironment, including tumour antigens and danger signals (also known as damage‐associated molecular patterns, DAMPs) [47]. These DAMPs can initiate inflammatory response, recruit immune cells and facilitate recognition of tumour cells. This process is known as immunogenic cell death (ICD) [48, 49, 50]. It has been shown that anthracyclines such as doxorubicin can induce ICD and thereby elicit a dendritic cell‐mediated tumour‐specific CD8^+^ T‐cell response in a colon carcinoma mouse model [51]. Moreover, doxorubicin was reported to selectively deplete myeloid‐derived suppressor cells from the tumour microenvironment, which relieved the immunosuppressive impact of these cells in a murine breast cancer model [52]. Recently, it is observed that the C‐type lectin receptor Clec2d is activated by binding histones to induce inflammation and tissue damage responses [53]. So it would be interesting to test whether histones can be externalized by doxorubicin, detected by the Clec2d receptor and cause an inflammation response. The immune stimulatory activity of doxorubicin, in the context of immune checkpoint blockade, was confirmed in a multi‐arm noncomparative phase II trial. Treatment of triple negative breast cancer patients with doxorubicin followed by PD1 blockade resulted in an overall response rate of 35%, compared with 17% for PD1 blockade alone [54]. Although this finding needs to be confirmed in larger cohorts, it suggests that the immune modulating function of anthracyclines may have a synergistic role in the overall anticancer activity in patients.

## Anthracycline‐associated severe side effects and preventive solutions

Although doxorubicin has been a cornerstone in cancer treatment for nearly five decades, its use is plagued with severe and treatment‐limiting side effects. Next to common generally acute and reversible chemo‐related adverse effects, such as nausea, vomiting, diarrhoea and bone marrow suppression, anthracycline treatment is associated with long‐term side effects, namely cardiotoxicity, therapy‐related malignancies and gonadotoxicity. These long‐term adverse effects severely impact the quality of life of cancer survivors, which limit the further application of anthracyclines. Therefore, extensive research has been performed to understand and reduce the anthracycline‐induced long‐term side effects.

### Cardiotoxicity

The most treatment‐limiting and therefore probably the best studied side effect of anthracyclines is cardiotoxicity. Anthracycline‐induced cardiotoxicity presents as cardiomyopathy, ventricular dysfunction, pericarditis‐myocarditis syndrome or arrhythmias and is dose‐dependent and irreversible [10, 55, 56]. As a result, doxorubicin treatment is limited to a cumulative dose of 450–550 mg·m^−2^ [9, 10]. Besides cumulative dose, the risk of cardiotoxicity is also associated with treatment schedule, age extremes, and combinations with other drugs or radiotherapy in the heart region [57, 58]. Currently, there is no management or medication to relieve anthracycline‐induced cardiotoxicity, and the only option for patients with severe symptoms is a heart transplantation. Therefore, doxorubicin is excluded from treating patients with a poor heart function, usually old patients. Thus, alleviating cardiotoxicity would greatly improve cancer treatment with anthracyclines.

Multiple mechanisms have been proposed, including mitochondrial dysfunction and/or lipid peroxidation as a result of ROS formation, targeting Topo II beta (Topo IIβ) in cardiomyocytes, and effects on calcium homeostasis [59, 60, 61, 62]. To reduce anthracycline‐induced cardiotoxicity, several attempts to manipulate these pathways have been made. In the following sections, we will discuss these in detail and propose a possible solution based on recent data.

### ROS alleviation

The most intensely studied mechanism of anthracycline‐induced cardiotoxicity is ROS production through interference with redox cycling and mitochondrial function [63]. To meet the high demand of ATP supply, cardiomyocytes have a greater density of mitochondria compared with other tissues, which could explain why the heart is more affected by anthracycline‐induced ROS production than other tissues [59, 64]. Green *et al*. [65] showed that doxorubicin‐induced mitochondrial dysfunction coincided with the production of ROS and cytochrome C release, which in turn activated Caspase‐3 and initiate apoptosis in H9C2 cardiac cells. It was reported that pretreatment with the free radical scavenger tocopherol reduced the cardiotoxicity of doxorubicin in a lymphoma mouse model, without affecting its antitumour efficacy [60]. Although similar results were observed in an AML animal model, the cardiac protective effects of radical quenchers in clinical trials were disappointing [66, 67].

Similar to ROS scavengers, most iron‐chelating agents can reduce ROS formation and alleviate doxorubicin‐induced cardiotoxicity in preclinical models. However, such benefits were not observed in patients [68, 69]. The iron chelator dexrazoxane is an exceptional case. It was reported to reduce anthracycline‐induced cardiotoxicity in some clinical studies, albeit not in all [70, 71]. However, this reduced toxicity is likely mediated by mechanisms different from ROS quenching, since other iron chelators are not cardiac protective [72]. Several alternative mechanisms of dexrazoxane function have been proposed, including inhibition of both apoptosis and necroptosis of cardiomyocytes [73] and antagonizing doxorubicin‐induced DNA damage by interfering with Topo IIβ [74].

Although it is convincingly shown that anthracyclines can induce ROS formation in *in vitro* studies, the discrepancy between the effectivity of ROS scavengers and iron chelators in preclinical studies and patients challenges the contribution of ROS production in anthracycline‐induced heart damage. Using appropriate preclinical cardiotoxicity models and treatment with anthracyclines at clinical relevant concentrations and schedules may help clarifying this issue.

### Precluding from targeting topoisomerase IIβ in cardiomyocytes

In human, Topo II enzymes are expressed in two isoforms, Topo IIα and Topo IIβ [75]. Although these two isoforms are encoded by different genes, they share substantial amino acid sequence identity and exhibit almost identical enzymological properties [76]. Notwithstanding their similarities, the expression patterns of Topo IIα and Topo IIβ are different. Topo IIα is mainly expressed in proliferating cells, and almost absent in quiescent and differentiated tissues. Topo IIα is associated with replication forks and stays bound to chromosomes during mitosis, which makes its expression essential for proliferation. On the contrary, Topo IIβ expression is independent of proliferation status and is high in most cell types [76]. In line with this notion, adult mammalian cardiomyocytes express Topo IIβ, but no detectable Topo IIα. Zhang *et al*. [61] reported that targeting Topo IIβ in cardiomyocytes by doxorubicin is important for the initiation of cardiotoxicity. It was shown that mice with cardiomyocytes‐selective conditional Topo IIβ knockout (Topo IIβ^+/Δ^ and Topo IIβ^Δ/Δ^) were not susceptible to the cardiac impairment caused by doxorubicin as observed in Topo IIβ^+/+^ mice. Further, Lyu *et al*. [74] reported that dexrazoxane reduced doxorubicin‐induced DNA damage in cardiomyocytes *in vitro* by rapid proteasomal degradation of Topo IIβ. These studies indicate that the DSBs mediated by Topo IIβ poisoning is a major cause of doxorubicin‐induced cardiotoxicity. Nevertheless, DSB cannot be the only reason, since the structurally nonrelated Topo II poison etoposide does not cause cardiotoxicity. From a clinical point of view, it suggests that Topo IIα‐specific anthracycline would prevent cardiotoxicity in patients and that Topo IIβ expression could be used as a prognostic marker for cardiotoxicity. Unfortunately, no genuine Topo IIα‐ or Topo IIβ‐specific drugs are available in clinic at present.

### Novel delivery strategies to reduce anthracycline‐induced toxicity

Due to the unsatisfactory effects of ROS scavengers and iron chelators in the clinic, tumour‐specific drug delivery systems were introduced in 1990s to reduce doxorubicin‐induced toxicities. These delivery strategies included nanoparticle encapsulated liposomal doxorubicin (LD) and pegylated LD (PLD). LD and PLD both show prolonged serum half‐life and a smaller volume of distribution compared with conventional doxorubicin [77]. LD and PLD can extravasate into the tumour via gaps in the micro vessels, whereas other tissues are much less permeable through tight junctions. Therefore, the long serum circulation of LD and PLD results in more specific tumour accumulation. Various animal models, as well as clinical trials, showed that these particles significantly decreased cardiotoxicity compared with conventional doxorubicin, without compromising antitumour efficacy [78, 79, 80]. Therefore, both LD and PLD are approved by the FDA for treating AIDS‐related Kaposi's sarcoma, multiple myeloma, breast and ovarian cancer, but their clinical application is limited by drug leakage and higher costs.

### Separating chromatin damage from DNA damage

With the aim to identify more effective anthracyclines with fewer side effects, thousands of doxorubicin analogues, either isolated from natural sources, produced by mutant enzymes or prepared by organic (semi)synthesis, have been evaluated in the past decades. However, only few variant drugs showed reduced cardiotoxicity without loss of anticancer activity. One such analogue which entered the clinic is epirubicin. In a meta‐analysis, epirubicin treatment showed significantly less cardiotoxicity compared with doxorubicin (OR, 0.39, 95% confidence interval: 0.20–0.78, *P* = 0.008) and subclinical cardiotoxicity (OR, 0.30, 0.16–0.57, *P* < 0.001) without compromising antitumour efficacy [81]. Therefore, epirubicin can be used at higher cumulative dose (900–1000 mg·m^−2^) compared with doxorubicin (450–550 mg·m^−2^). Although epirubicin can be used at higher cumulative dose, its application is still limited by cardiotoxicity.

**Fig. 4:**
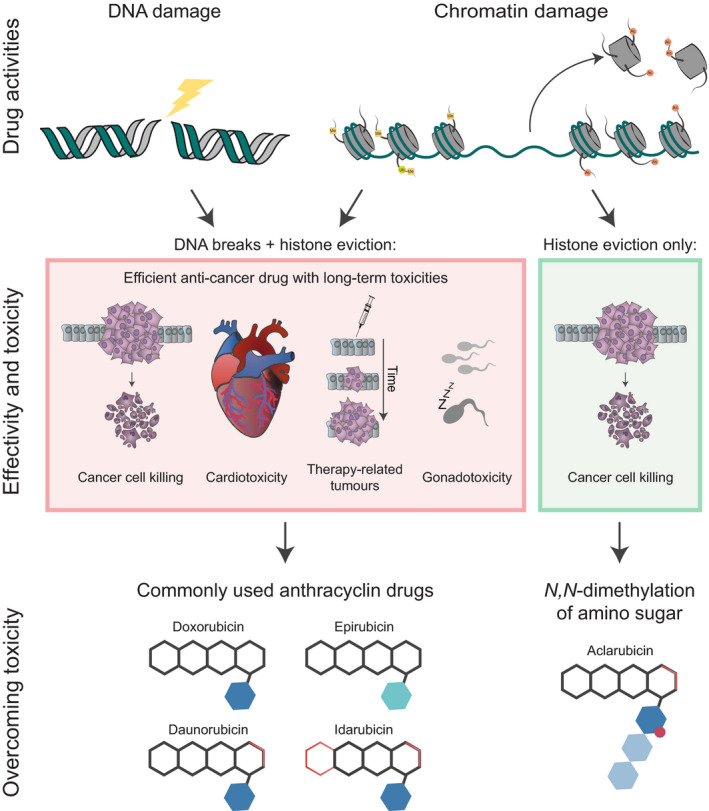
Schematic overview of the activities and toxicities of the clinically used anthracyclines and their underlying mechanisms. Most commonly used anthracyclines, including doxorubicin, epirubicin, daunorubicin and idarubicin, possess both DNA‐ and chromatin‐damaging activities. As a consequence, these drugs are associated with cardiotoxicity, therapy‐related malignancies and gonadotoxicity. *N,N*‐dimethylation of the sugar moiety, as for aclarubicin (and diMe‐doxorubicin), results in anthracycline variants with only chromatin‐damaging activity, which are effective anticancer drugs with limited toxicities.

The key question for the development of analogues with reduced toxicity is whether these toxic effects and anticancer activities are mediated by the same mechanism(s), which determines whether it is theoretically feasible to eliminate the cardiotoxicity of anthracycline without compromising its therapeutic efficacy. Recent work of our group provides some insight. We observed that aclarubicin, as well as the doxorubicin analogue diMe‐doxorubicin, showed strongly reduced cardiotoxicity in various mouse models and human‐induced pluripotent stem cells‐derived cardiomyocyte microtissues, without compromising anticancer activity [44]. *N,N*‐dimethylation of the amino sugar eliminated the DNA‐damaging capacity of these compounds, while retaining effective histone eviction activity (Fig. 4). On the other hand, etoposide and amrubicin, with only DNA‐damaging activity, are also not cardiotoxic in mouse models and patients, but display much lower anticancer activity. These observations indicate that the combination of DNA with chromatin damage, as for doxorubicin and other clinically used anthracyclines, is responsible for the cardiotoxicity of these drugs [44]. Therefore, variants with only chromatin‐damaging activity would be a promising direction for the development of next‐generation anthracyclines. Furthermore, the identification of the structure–activity relationship of the sugar moiety and cardiotoxicity provides a new strategy for anthracycline development.

### Therapy‐related malignant neoplasms

Attributing to the increased survival of cancer patients which modern anticancer therapy has made possible, the long‐term side effects, such as tumorigenicity, have become an issue. Currently, 17–19% of all new primary malignancies occur in cancer survivors [82, 83]. Among all the long‐term adverse effects caused by chemotherapy, therapy‐related malignant neoplasms (t‐MNs) are one of the most deleterious, because of substantial morbidity and considerable mortality. Soon after discovery, anthracyclines (excluding aclarubicin hereafter in this section) has been found to cause transformation and mutagenesis *in vitro* and tumorigenic *in vivo* [84, 85, 86, 87, 88, 89, 90], and anthracycline exposure is associated with increased risks of t‐MNs in cancer survivors. The t‐MNs most often ascribed to anthracyclines are AML [91, 92, 93], sarcoma [94, 95, 96] and female breast cancer [96, 97]. Thyroid cancer [98] and acute promyelocytic leukaemia (APL) [99, 100] have also been linked to antecedent anthracycline treatment.

The anthracycline therapy‐related AMLs (t‐AMLs) frequently exhibit balanced chromosomal translocations at 11q23 (involving *MLL1* gene) or 21q22 (involving *AML1/RUNX1/CBFA2* gene), however occurring at unique breakpoints than *de novo* AML with the same cytogenetics [101, 102, 103, 104]. In contrast to alkylating agent‐associated t‐AMLs, these leukaemias are rarely preceded by a myelodysplastic phase [105]. They develop with a shorter latency, often within 1–3 years after the initial anthracycline‐based chemotherapy and, in some cases, within 1 year [106]. Due to unfavourable, complex or monosomal karyotypes, these t‐AMLs often present as aggressive diseases and are associated with poor prognosis compared with *de novo* AML [106, 107]. Anthracyclines are also involved in the development of therapy‐related APL (t‐APL) featured with balanced translocation of t(15;17) [99], which results in a double dominant‐negative fusion protein, PML‐RARα [100]. Anthracycline‐associated t‐APL also arises after a short latency period, usually without a preleukaemic phase [99, 100]. After a peak at 2 years following primary anthracycline treatment, the incidence of t‐APL quickly decreases with time. Although the chromosomal breakpoints induced by anthracyclines are distinct from those observed in *de novo* t(15;17) APL, the clinical outcomes of t‐APL and *de novo* APL are similar after all‐trans retinoic acid‐ and anthracycline‐based treatments, for which the 5‐year survival rate is about 80% [99, 100]. Anthracycline‐associated solid tumours typically occur >10 years after exposure and in a dose‐dependent manner [94, 95, 96, 97, 98]. There is not much known about the genetic alterations of anthracycline‐related solid tumours, though a strong dose–response correlation with doxorubicin was found in survivors of Li‐Fraumeni syndrome‐associated cancer types compared with other childhood cancer survivors [96]. Furthermore, we recently reported that doxorubicin single drug treatment induced breast cancer development in *Trp53^+/−^* female mice, indicating the direct contribution of doxorubicin treatment to tumour development [44].

Besides the tumorigenicity of anthracyclines, cancer survivors may be especially susceptible to developing t‐MNs due to a variety of other risk factors. These include genetic predisposition (such as the abovementioned Li‐Fraumeni syndrome), carcinogenic exposures in common (such as tobacco use or alcohol abuse), host effects (age, gender, immunodeficiency or obesity) and combination therapy with other mutagenic chemotherapeutics (alkylating agents, etoposide or radiotherapy) [82, 83, 95, 96, 97]. Therefore, the exact mechanisms how anthracyclines contribute to t‐MN development remain unclear. One option follows reports showing that leukaemia‐associated translocation t(8;21) can be detected in hematopoietic cells of healthy individuals with no overt leukaemia [108, 109], and anthracycline‐related t(8;21) t‐AMLs were found to be positive for JAK2 V617F mutation [110], which suggests that t‐AML is the consequence of a series of genetic alterations. Anthracyclines may facilitate the complete transformation of preleukaemic cells by introducing additional mutations. On the other hand, anthracyclines can cause chromosomal translocations through an indirect mechanism mediated by apoptotic nucleases [111, 112, 113]. Nevertheless, accumulating evidence suggests that anthracyclines play a direct role in causing t‐MN associated genetic aberrations. Anthracyclines generate DSBs by hijacking Topo II, particularly at breakpoint hotspot regions of leukaemic translocations [103]. Unfaithful repair by error‐prone DNA repair pathways can then result in mutagenesis or chromosomal translocations [114]. Through a similar mechanism of action, the structurally unrelated Topo II poison etoposide was also found to be associated with t‐MNs of similar karyotypes in a dose‐dependent manner, albeit less potent than anthracyclines [95, 115, 116]. The inferior potency of etoposide in transformation is also observed in a *Trp53^+/−^* mouse model treated with single agents of comparable dose and schedule, which excluded the influence of genetic predisposition of host and concurrent anticancer therapies [44]. This tumorigenic difference can be explained by the strongly delayed DNA repair of anthracycline due to eviction of histone variant H2AX [27].

H2AX is an important histone variant for DNA damage repair, which is phosphorylated at DNA damage sites and responsible for repair machinery recruitment. Eviction of H2AX by doxorubicin greatly attenuates DNA damage repair and consequently results in enhanced cell death and more transformation compared with etoposide [27, 43]. In line with this hypothesis, the same *Trp53^+/−^* mouse experiment and *in vitro* data showed that aclarubicin and diMe‐doxorubicin without DNA‐damaging activity are not tumorigenic [44, 117, 118]. Collectively, DNA damage induced by Topo II poisons is a main cause of t‐MNs.

As mentioned above, anthracyclines evict histones with different epigenomic selectivity. It is interesting to notice that t(11q23) AML with *MLL1* translocation is also associated with epigenetic changes, since MLL1 is an H3K4 methyltransferase [119]. The C‐terminal SET domain of MLL1, which is responsible for methylating H3K4, is missing in the fusion oncoprotein of 5′‐*MLL1–partner*‐3′ rearrangement. Epigenetic profiling after MLL1 deletion or with MLL1 fusion proteins revealed reduced H3K4 methylation at promotor region of target genes [120, 121]. Considering the selectivity of doxorubicin for H3K4me3 at active promotors, this coincidence may provide another explanation for the development of t(11q23) AML and its resistance to doxorubicin‐based regimens [122, 123]. As a result, anthracyclines with different histone eviction profiles, such as aclarubicin and diMe‐doxorubicin, could provide alternative treatment options for doxorubicin‐resistant AMLs, and vice versa [45, 124, 125, 126, 127, 128].

Due to limited understanding of the mechanisms of action, t‐MN was previously considered as the original sin of anthracycline treatment because of resulted DNA damage. Hence, hope was laid on early detection of t‐MNs by intense follow‐up screening in susceptible cancer survivors or restraint of high cumulative dose of anthracyclines. However, the discovery of histone eviction activity of anthracyclines not only offers a new anticancer mechanism, but also provides a strategy to prevent t‐MNs, which is experimentally illustrated by aclarubicin [44, 117]. The recent understanding on the structure–activity relationship of anthracyclines makes it possible to eliminate the DNA‐damaging activity of anthracycline and related toxicities, while remaining their anticancer efficacy.

### Gonadotoxicity

Owing to its mechanisms, doxorubicin also targets healthy tissues with high proliferating rates, such as myeloid and lymphoid tissues, gastrointestinal mucosa and gonads. Since the survival rates of cancer patients improved spectacularly in the last two decades, the number of cancer survivors suffering from doxorubicin‐induced gonadotoxicity also strongly increased [129]. Gonadotoxicity not only causes psychosocial distress, but also increases the risk of subsequent complications, such as osteoporosis, infertility and cardiovascular disease [130]. Gonadal damage caused by doxorubicin treatments happens to patients at all stages of life. Although many of the cancer survivors could regain gonadal functions in a few months or years after doxorubicin treatment [131], they may have a shortened reproductive lifespan or late effects on pregnancy than the age‐matched normal population [132, 133, 134]. Currently, cryopreservation of gametes or embryos is the only option to preserver fertility in patients receiving doxorubicin‐containing therapy. However, this approach is only applicable to patients in a reproductive age and can be problematic in adolescent patients. For patients who have not yet commenced puberty, there is no clinically approved method for fertility preservation at present [135], despite that previous doxorubicin treatment during prepubertal period can lead to severe injury of the adult fertility [136].

Several classes of compounds have been proposed to protect gonads from doxorubicin insult in mouse models, including hormone agonists [137], anti‐oxidants [138, 139], proteasome inhibitors [140], tyrosine kinase and DDR inhibitors [141]. Before validating these drugs in a patient cohort, it is more important to test whether these inhibitors alleviate the gonadotoxicity without compromising the anticancer activity of doxorubicin *in vivo*. Nevertheless, development of active anthracycline variants with limited gonadotoxicity would be a preferable strategy, if possible. The depletion of follicular reserve in females and depletion of spermatogenesis in males caused by doxorubicin treatment can be attributed to the DSBs generated by the drug and subsequent cell death of germ cells [134, 142, 143, 144]. Besides direct germ cell destruction, doxorubicin also causes DSBs in somatic cells, vasculature and apoptosis of the stromal compartments in gonads [136, 143, 145, 146, 147]. The latter then further impairs the development of fertile germ cells. Similar effects were also observed for the nonanthracycline Topo II poison etoposide, which also causes DSBs and destruction of gonads [148, 149]. These data suggest that the DNA‐damaging activity of doxorubicin plays an important role in mediating gonadotoxicity. This observation is further strengthened by our recent study showing that aclarubicin and diMe‐doxorubicin, both lacking DNA‐damaging activity but with comparable antitumour capacity as doxorubicin, did not cause apoptosis of developing follicles in female mice [44]. However, diMe‐doxorubicin, with different histone eviction profile than aclarubicin and doxorubicin (unpublished results), still induced depletion of spermatogenesis in male mice, albeit at a lower degree than doxorubicin.

Oxidative stress has also been proposed as a mechanism of doxorubicin‐induced gonadotoxicity [150]. However, some work using spermatogonia and immature Sertoli cell lines has shown no increase of ROS formation before the onset of cytotoxicity [151]. In line with this observation, co‐administration of anti‐oxidants showed no protective effect on doxorubicin‐induced testicular toxicity *in vivo* [139, 152]. Collectively, these data suggest that DNA‐damaging activity of doxorubicin is a major cause for gonadotoxicity, especially in females, with perhaps some contribution of specific histone eviction in the case of diMe‐doxorubicin in male gonadotoxicity.

## Perspectives

Since the discovery of daunorubicin and doxorubicin in the 1960s, a search for less toxic yet effective alternatives to doxorubicin was initiated in the 1980s. Out of thousands of anthracycline variants tested, only a few entered the clinic, most notably epirubicin, idarubicin and aclarubicin. One reason for this limited number of successful compounds might be the lack of consensus on the mechanism of action of anthracyclines for their anticancer activity and toxicities. Furthermore, whether the severe toxicities of these drugs are intimately connected with their anticancer activity has been a lingering topic in the field. For a long time, DSB induction was considered as the main anticancer activity of anthracyclines. While only recently, a second activity –chromatin damage as a result of histone eviction– was proposed [27, 39]. Chromatin damage is not only a novel activity of anthracyclines, but also a new anticancer mechanism, which is not found in other types of chemotherapeutics. The ground‐breaking discovery of chromatin damage is granted by modern molecular technologies, such as time‐lapse confocal imaging, photoactivation and various next‐generation sequencing techniques. Hence, it is still meaningful to re‐investigate old drugs with modern technology. This may yield new mechanisms of action that can be explored to arrive at active and detoxified doxorubicin and other drug variants. Additionally, this resulted in the rediscovery of an anthracycline variant, aclarubicin, as a less toxic but very active drug in (relapsed) AML treatment.

While the potential cardiotoxicity‐low/free anthracyclines need to be tested in clinic, some improvements of current anthracycline‐containing chemotherapy regimen should be considered. For instance, it would be debatable to combine anthracyclines with etoposide in the same treatment regimen concerning the contribution of DNA‐damaging activity to multiple toxicities, although this is frequently used in AML treatment. Likewise, specific anthracycline variant should be carefully selected for children cancer patients or patients with predisposal genetic disorder to avoid toxicities. The new mechanism, histone eviction with certain (epi)genomic selectivity, indicates that anthracyclines are in fact also epigenetic drugs. Preliminary data showed that diffuse large B‐cell lymphoma cells with elevated levels of H3K27me3 were more susceptible to aclarubicin than daunorubicin [43], indicating anthracycline variant selection can be personalized for cancer treatment based on their histone eviction profiles.

The recent understanding on anthracycline anticancer activity and toxicities suggests that anthracycline development should focus on depleting DNA‐damaging activity from chromatin‐damaging activity. Such drugs should allow effective anthracycline‐based therapies devoid of the major treatment‐limiting adverse effects: cardiotoxicity, therapy‐related malignancies and gonadotoxicity. This would especially benefit cancer patients with a poor heart function, which are currently excluded from anthracycline‐based chemotherapy. In addition, drug variants lacking these side effects could be used in more intense and/or longer therapy and could be used for relapsed patients with a history of anthracyclines‐based therapies.

In conclusion, despite the long history of anthracyclines, the novel discovery of chromatin damage as the major antitumour activity and its collective contribution with DNA‐damaging activity to toxicities, allows the development of potentially new treatment strategies to improve cancer therapy and the quality of life of cancer survivors.

## Conflict of interest

JN is a shareholder in NIHM that aims to produce aclarubicin for clinical use.

## Author contributions

SvdZ and XQ conceived the manuscript and constructed the text under supervision of JN.

### Peer Review

The peer review history for this article is available at https://publons.com/publon/10.1111/febs.15583.
